# Melatonin Receptor Agonists as the “Perioceutics” Agents for Periodontal Disease through Modulation of *Porphyromonas gingivalis* Virulence and Inflammatory Response

**DOI:** 10.1371/journal.pone.0166442

**Published:** 2016-11-10

**Authors:** Wei Zhou, Xuan Zhang, Cai-Lian Zhu, Zhi-Yan He, Jing-Ping Liang, Zhong-Chen Song

**Affiliations:** 1 Department of Periodontology, Ninth People’s Hospital, Shanghai Jiao Tong University School of Medicine, Shanghai Key Laboratory of Stomatology, 639 Zhi Zao Ju Road, Shanghai 200011, China; 2 Shanghai Research Institute of Stomatology, Ninth People’s Hospital, Shanghai Jiao Tong University School of Medicine, Shanghai Key Laboratory of Stomatology, 639 Zhi Zao Ju Road, Shanghai 200011, China; 3 Department of Pharmacy, Ninth People’s Hospital, Shanghai Jiao Tong University School of Medicine, 639 Zhi Zao Ju Road, Shanghai 200011, China; Taipei Medical University, TAIWAN

## Abstract

**Aim:**

“Perioceutics” including antimicrobial therapy and host modulatory therapy has emerged as a vital adjunctive treatment of periodontal disease. Melatonin level was significantly reduced in patients with periodontal diseases suggesting melatonin could be applied as a potential “perioceutics” treatment of periodontal diseases. This study aims to investigate the effects of melatonin receptor agonists (melatonin and ramelteon) on *Porphyromonas gingivalis* virulence and *Porphyromonas gingivalis*-derived lipopolysaccharide (Pg-LPS)-induced inflammation.

**Methods:**

Effects of melatonin receptor agonists on *Porphyromonas gingivalis* planktonic cultures were determined by microplate dilution assays. Formation, reduction, and viability of *Porphyromonas gingivalis* biofilms were detected by crystal violet staining and MTT assays, respectively. Meanwhile, biofilms formation was also observed by confocal laser scanning microscopy (CLSM). The effects on gingipains and hemolytic activities of *Porphyromonas gingivalis* were evaluated using chromogenic peptides and sheep erythrocytes. The mRNA expression of virulence and iron/heme utilization was assessed using RT-PCR. In addition, cell viability of melatonin receptor agonists on human gingival fibroblasts (HGFs) was evaluated by MTT assays. After pretreatment of melatonin receptor agonists, HGFs were stimulated with Pg-LPS and then release of cytokines (IL-6 and lL-8) was measured by enzyme-linked immunosorbent assay (ELISA).

**Results:**

Melatonin and ramelteon did exhibit antimicrobial effects against planktonic culture. Importantly, they inhibited biofilm formation, reduced the established biofilms, and decreased biofilm viability of *Porphyromonas gingivalis*. Furthermore, they at sub-minimum inhibitory concentration (sub-MIC) concentrations markedly inhibited the proteinase activities of gingipains and hemolysis in a dose-dependent manner. They at sub-MIC concentrations significantly inhibited the mRNA expression of virulence factors (*kgp*, *rgpA*, *rgpB*, *hagA*, and *ragA*), while increasing the mRNA expression of ferritin (*ftn*) or hemolysin (*hem*). They did not show obvious cytotoxicity toward HGFs. They inhibited Pg-LPS-induced IL-6 and IL-8 secretion, which was reversed by luzindole, the melatonin receptor antagonist.

**Conclusion:**

Melatonin receptor agonists can inhibit planktonic and biofilm growth of *Porphyromonas gingivalis* by affecting the virulent properties, as well as Pg-LPS-induced inflammatory response. Our study provides new evidence that melatonin receptor agonists might be useful as novel “perioceutics” agents to prevent and treat *Porphyromonas gingivalis*-associated periodontal diseases.

## Introduction

Periodontal diseases have a global distribution and all types of periodontal disease are infectious disorders resulting from the interplay between pathogenic agents and host immune reactions [1]. “Perioceutics” is addressed the use of pharmacotherapeutic agents including antimicrobial therapy and host modulatory therapy, specifically developed to manage periodontal disease [2–4]. “Perioceutics” has emerged as a vital adjunctive treatment of periodontal disease [3,4]. Hence, the development of the “perioceutics” agents is of great significance in the better management of periodontal disease.

Melatonin (N-acetyl-5-methoxytryptamine) is a well-studied, endogenous molecule that regulates and modulates a wide variety of physiological functions. Besides its important role as a chronobiotic, melatonin has been found to be involved in a variety of pathophysiological processes including the modulation of immune response, anti-inflammatory, antioxidant, antitumoral, neuroprotective processes [5–7].

Recent studies have shown that melatonin level was significantly reduced in patients with periodontal diseases, suggesting melatonin be applied as an important biomarker in the diagnosis and also potential treatment of periodontal diseases [7–11]. A cross-sectional study has demonstrated that topical application of melatonin on gingival tissues has a favorable effect on preventing the periodontal disease [12]. In vivo studies were successfully performed with melatonin in periodontal disease models indicating that melatonin could exert protective and preventive effects [13–15]. Choi et al. reported that melatonin inhibited Prevotella intermedia LPS-induced inflammation [16].

As is well known, microbial plaque has been recognized as the primary etiology for the development of periodontal disease [17,18]. Among the oral microbiota in periodontal disease, *Porphyromonas gingivalis*, as a Gram-negative, rod-shaped, and anaerobic bacterium, is strongly implicated as a “keystone” periodontal pathogen [1,19,20]. *Porphyromonas gingivalis* utilizes multiple virulence factors, such as proteinases (e.g. gingipains), fimbriae, LPS, and cytotoxic and hemolytic molecules [21–24]. Several genes involved in virulence of *Porphyromonas gingivalis* have been identified, for example, *kgp*, *rgpB*, *vimA*, *ragA*, and hemagglutinin (*hagA*), et al [24]. Activity of these virulence factors is critical and essential for the growth and survival of *Porphyromonas gingivalis* as nutrients. Virulence factors are helpful to adheres to lots of host cell surfaces and play vital roles in the breakdown of host-defense mechanisms, the penetration and progressive damage of the host connective tissues [25,26]. Furthermore, virulence factors such as LPS, can stimulate the production of inflammatory factors, for example interleukins (IL) [26], which are involved in the activation and development of inflammation in periodontal pockets.

To date, only a few involve the in vitro antimicrobial activities of melatonin in infectious diseases, and its effect remains controversial. For example, Elmahallawy et al. and Schuck et al. reported that melatonin exhibited anti-parasite activity [27,28]. Tekbas reported that melatonin exerted a potent antimicrobial activity on multidrug-resistant, gram-positive and gram-negative bacteria [29]. Ozturk et al. reported that 300 μg/mL melatonin inhibited *Candida albicans* [30]. Conversely, Wang et al. and Konar et al. performed in vitro antimicrobial studies of melatonin and demonstrated that antimicrobial properties of it are limited [31,32]. Gomez-Florit reported that melatonin did not affect *Staphylococcus epidermidis* growth [8]. However, there is no report on the antimicrobial activity of melatonin against oral pathogens.

Ramelteon, a melatonin derivative, was the first melatonin receptor agonist approved for human use. In 2005, the US Food and Drug Administration (FDA) licensed it in the USA for the treatment of insomnia [33]. Compared to melatonin, ramelteon has a higher lipophilicity and it is more easily taken up and retained by tissues, so ramelteon can be of much therapeutic value. Therefore, the present study aims at evaluating the “perioceutics” therapeutic potential of melatonin receptor agonists (melatonin and ramelteon) on antimicrobial activity and anti-inflammatory action against *Porphyromonas gingivalis*, a “keystone” periodontal pathogen.

## Materials and Methods

### 2.1 Chemicals, bacteria and culture conditions

Commercial melatonin was purchased from Sigma-Aldrich Corp. (St. Louis, MO, USA). Ramelteon was purchased from Selleck Chemicals (Houston, TX, USA) and luzindole was purchased from Tocris Bioscience (Bristol, UK). Test samples were prepared by dissolving melatonin and ramelteon in dimethyl sulfoxide (DMSO), and the indicated concentration was reached through dilution with Tryptic Soy Broth (TSB; Bacto^™^, BD, Sparks, MD, USA) or Dulbecco’s Modified Eagles Medium (DMEM, Hyclone, Logan, UT, USA). DMSO was used as the vehicle control in the experiments. The test bacterium *Porphyromonas gingivalis* ATCC33277 was provided by the Shanghai Research Institute of Stomatology and Shanghai Key Laboratory of Stomatology, Shanghai Ninth People’s Hospital, Shanghai Jiao Tong University School of Medicine (Shanghai, China) and grown in fresh TSB at 37°C under anaerobic conditions (80% N_2_, 10% H_2_, 10% CO_2_).

### 2.2 Bacteria antibiotic susceptibility assay

The drug susceptibility of planktonic cultures was determined by a 2-fold serial dilution method [29]. Dilutions of melatonin (3.13–1600 μg/mL) and ramelteon (3.13–800 μg/mL) were prepared in TSB, and added in a flat-bottomed 96-well microplate. A 20 μL quantity of Porphyromonas gingivalis suspension at a final concentration of 1×10^7^ colony-forming units (CFU)/mL was added and incubated under anaerobic conditions for 48 h at 37°C. Control wells that were not inoculated or that contained the vehicle control were also prepared. Bacterial growth was monitored at the wavelength of 630 nm. The MIC referred to the lowest concentration of melatonin at which inhibited growth to a level of < 0.05 at 625 nm, i.e. no macroscopically visible growth [29].

To determine the minimal bactericidal concentration (MBC) values, an aliquot of 10 μl cell suspension from each well was taken, and bacterial clones were counted after incubation for 3–5 days at 37°C under anaerobic conditions. MBC referred to the lowest concentration at which any colony did not grow on the agar.

### 2.3 Biofilm susceptibility assay

The effects on Porphyromonas gingivalis biofilm formation were examined by the crystal violet staining method [34,35]. A 200 μl aliquot of Porphyromonas gingivalis suspension (1×10^7^ CFU/mL) was grown in TSB supplemented with drugs for 48 h at 37°C in a 96-well microplate. The culture supernatant was removed and the adherent biofilms were rinsed for three times using phosphate buffered saline (PBS). Then, the adherent biofilms were incubated with methanol for 15 min followed by staining with 0.04% (w/v) crystal violet for 15 min. After washing with deionized water, the microplate was dried overnight. Finally, 95% ethanol was used to dissolve and the microplates were shaken for 1 h at room temperature. The OD values were recorded at wavelength of 550 nm. Definition of the minimum biofilm inhibition concentration (MBIC) was the lowest drug concentration at which at least 50% of biofilms was inhibited compared with the control (MBIC_50_) [34].

The effects of melatonin and ramelteon on mature *Porphyromonas gingivalis* biofilm reduction were examined by the crystal violet staining method [34,35]. A 200 μL *Porphyromonas gingivalis* suspension (1×10^7^ CFU/mL) was added to the 96-well microplate so as to develop the biofilms. The culture medium was decanted carefully with the integrity of biofilms under anaerobic incubation for 48 h at 37°C. The formed biofilms were gently rinsed with PBS and non-adherent cells were removed. Bacterial cells were incubated in the presence of drugs for 24 h. Then, the media were removed and biofilms were gently washed with PBS and subsequently fixed with methonal. The biofilms were stained with 0.04% crystal violet and recorded using a microplate reader. The minimum biofilm reduction concentration (MBRC) was the lowest drug concentration at which at least a 50% (MBRC_50_) of the biofilms were reduced compared with the control [34].

The effect of melatonin and ramelteon on the viability of in vitro *Porphyromonas gingivalis* biofilms were determined using the 3-(4,5-dimethylthiazolyl-2)-2,5-diphenyltetrazoliumbromide (MTT) method modified from that of Tang HJ et al [36]. The *Porphyromonas gingivalis* biofilm formation, incubation of the biofilm and the drugs were the same as described for the above method. MTT (0.5 mg/mL) was added to detect the metabolic activity of the biofilms. All plates were cultured in the dark for 2 h at 37°C. Following incubation, MTT solution was gently aspirated from each well, and 100 μL of DMSO was added to dissolve the formazan crystals. After the plate was shaken for 10 min at room temperature in the dark, absorbance at 490 nm was recorded using a microplate reader. The sessile MIC (SMIC_50_) was the lowest drug concentration at which there was at least a 50% reduction compared with that of the control [34].

### 2.4 Biofilm measurement by CLSM

Porphyromonas gingivalis biofilms, grown as described above assay of biofilm formation for 48 h on glass coverslips, were formed in the presence or absence of sub-MIC drugs. Bacterial cells were stained with the LIVE/DEAD^®^ BacLight^™^ Bacterial Viability Kit (Molecular Probes Inc., Eugene, OR, USA). After staining for 15 min out of the light, CLSM (Leica TCS SP2; Leica DMIRE2 Microsystems, Wetzlar, Germany) was applied to acquire images and thickness of biofilm was measured. Live bacteria showed fluorescent green, whereas dead bacteria were fluorescent red. The exciting laser intensity, background level, contrast and electronic zoom were fixed for each experiment. In each experiment, at least four random fields were recorded.

### 2.5 Gingipain activity assays

The effect of melatonin and ramelteon on gingipain activity of Porphyromonas gingivalis was measured according to the previous methods [34,37]. Briefly, a 24 h Porphyromonas gingivalis culture (1×10^8^ CFU/mL) in the presence of sub-MIC levels of drugs was centrifuged (10,000 ×g for 15 min at 4°C), washed and suspended in PBS at an OD630 of 2 for Porphyromonas gingivalis Arg-gingipains (Rgp) activity and OD630 of 1 for Porphyromonas gingivalis Lys-gingipain (Kgp) activity. The Porphyromonas gingivalis cells were incubated with PBS with or without drugs at sub-MIC concentrations of drugs, a specific substrate for Rgp (0.4 mM, benzoyl-arginine-p-nitroanilide, BAPNA) or Kgp (0.4 mM, N-p-tosyl-glycine-proline-lysine-p-nitroanilide, TGPpNA), and a reductant (1 mM, DL-Dithiothreitol, DTT). The mixtures were incubated at 37°C in the dark. Activity of Rgp and Kgp indicated as the hydrolysis of the Rgp- and Kgp-specific chromogenic substrates was detected at wavelength of 405 nm. Substrates with drugs and substrates alone were individually used as controls. A 100% value was defined as the degradation after a 4 h-treatment without drugs.

### 2.6 Hemolytic activity assay

Hemolytic activity was performed as previously reported [38,39]. In brief, Porphyromonas gingivalis cells were centrifuged (10,000 × g for 10 min), washed three times with PBS, and then re-suspended to a final OD600 of 1.5. At the same time, sheep erythrocytes were harvested by centrifugation (4,400 × g for 20 min) and washed with PBS until the supernatant did not contain hemoglobin pigment visibly. The sheep erythrocytes at a concentration of 1% were suspended in PBS and then mixed with an equal volume of bacterial cells with or without drugs at sub-MIC concentrations of drugs at 37°C for 4 h. Then samples were further spun at 1,300 × g for 5 min and the supernatant was detected. The hemolytic activity was determined at wavelength of 405 nm. Erythrocytes were used alone as a negative control. Complete erythrocytes lysis was obtained by mixing 10 μL of a 10% (w/v) sodium dodecyl sulfate (SDS) solution with 1% (v/v) sheep erythrocytes. Relative hemolytic activity was determined compared to the vehicle group.

### 2.7 Quantitative analysis of gene expression by RT-PCR

The total RNA of Porphyromonas gingivalis was extracted by a Bacterial RNA Kit (Omega Bio-Tek, Norcross, GA, USA), and measured by Nanodrop2000 to determine the RNA concentration. Reverse transcription was performed by M-MLV Reverse Transcriptase Kit (Invitrogen, Carlsbad, CA, USA) to generate cDNA. Amounts of mRNA transcripts were measured by the Roche LightCycler 480 real-time PCR detection system (Roche, Basel, Switzerland). The *galE* gene was used as the housekeeping amplicon [40]. Reactions were performed with 20 μL of a mixture containing 10 μL of SYBR Premix Ex Taq II (2×; Takara), 1.6 μL of each gene-specific primer, 3.4 μL of sterile distilled water and 5 μL of the cDNA template. The forward and reverse primer sequences are shown in Table 1 [40,41]. Real-time PCR conditions included 30 s at 95°C; 10 s at 95°C, 20 s at 60°C and 15 s at 72°C for 40 cycles. Melting temperature curve analyses were used to validate the specificity of each primer pair. Data were analyzed using the comparative Ct method.

**Table 1:** The primers sequence of genes used in RT-PCR.

Target gene	Description	Primer sequence
*rgpA*	arginine-specific cysteine proteinase RgpA	Forward:5’-CACCGAAGTTCAAACCCCTA-3’
		Reverse:5’-GAGGGTGCAATCAGGACATT-3’
*rgpB*	arginine-specific cysteine proteinase RgpB	Forward:5’-GCTCGGTCAGGCTCTTTGTA-3’
		Reverse:5’-GGGTAAGCAGATTGGCGATT-3’
*kgp*	lysine-specific cysteine proteinase Kgp	Forward:5’-AGGAACGACAAACGCCTCTA-3’
		Reverse:5’-GTCACCAACCAAAGCCAAGA-3’
*hagA*	hemagglutinin protein HagA	Forward:5’-TAAATAAGGGCGGAGCAAGA-3’
		Reverse:5’-GACGGAAAGCAACATACTTCG-3’
*ragA*	receptor antigen A	Forward:5’-CGCTATTCTTCCTTTGCTTGCT-3’
		Reverse:5’-GATCGTGGTGTTTCCGACAA-3’
*vimA*	virulence modulating gene A	Forward:5’-TCGCGTAGTCTGAGAGTAACCTT-3’
		Reverse:5’-GGTATAAACGAAGACAGCACGAC-3’
*ftn*	ferritin	Forward:5’-CGGCGAGGTGAAGATAGAAG-3’
		Reverse:5’-CTCCTGAGAGAGACGGATCG-3’
*hem*	hemolysin	Forward:5’-ACGAAGCCTTGTTCTCCTCA-3’
		Reverse:5’-CAATGAATATGCCGGTTTCC-3’
*hmuR*	TonB-dependent receptor HmuR	Forward:5’-CTCCCATGCGGCCAACCCTCC-3’
		Reverse:5’-GCAGACGGGCTGTACGGCTACC-3’
*luxS*	S-ribosylhomocysteine lyase	Forward:5’-GAATGAAAGAGCCCAATCG-3’
		Reverse:5’-GTAATCGCCTCGCATCAG-3’
*galE*	UDP-glucose 4-epimerase	Forward:5’-TCGGCGATGACTACGACAC-3’
		Reverse:5’-CGCTCGCTTTCTCTTCATTC-3’

### 2.8 Ethics statement, cell culture and cytotoxicity assay

This study was approved by the Ethics Committee of the Shanghai Ninth People’s Hospital, Shanghai Jiao Tong University School of Medicine in May, 2015 (Approval Number: 2015–41). The forms of informed consents were obtained from the participants prior to taking part in this research.

Primary cultures of HGFs were excised from human gingival tissue. Gingival tissue explants were obtained from patients undergoing crown-lengthening surgery at the Shanghai Ninth People’s Hospital, Shanghai Jiao Tong University School of Medicine in June, 2015. Gingival specimens were washed in 70% ethanol, and then in 100 units/mL penicillin G and 100 μg/mL streptomycin. They were cut into small pieces and maintained in DMEM (Gibco, GrandIsland, NY, USA) supplemented with 15% heat-inactivated fetal bovine serum, 100 U/mL of penicillin G and 100 μg/mL of streptomycin at 37°C in 5% CO_2_ and humidified air. The cells were used for the experiments between passage 2 and passage 10.

The cell culture medium of HGFs was then replaced by serial dilutions of melatonin and ramelteon, with final concentrations ranging from 1 μg/mL to 400 μg/mL. DMEM without drugs served as a negative control. After 24 h of culture, MTT dye (0.5 mg/mL) was added and then incubated for 4 hours at 37°C. Following incubation, MTT was aspirated from each well, and 100 μL of DMSO was added to dissolve the formazan crystals. The plate was then shaken for 10 min at room temperature. The optical density (OD) values for each well were measured at 490 nm using a microplate reader (ELx800, Biotek Instruments, Winooski, VT, USA). A higher absorbance means a higher formazan concentration, indicating higher metabolic viability of HGFs in the wells.

### 2.9 Measurement of IL-6 and IL-8

HGF cells were treated with melatonin receptor agonists for 6 h, followed by Pg-LPS (InvivoGen, San Diego, USA) at 1 μg/mL concentration for 24 h. The release of IL-6 and IL-8 into the supernatant was measured using the commercial ELISA kits (Xi’tang biotechnology, shanghai, china), according to the manufacturer’s instructions.

### 2.10 Statistical analysis

Data were presented as the means ± SEM. Statistical analysis was performed with the Student t-test and a one-way analysis of variance (ANOVA) followed by Dunnett’s post hoc multiple comparison test. The difference was considered statistically significant at P < 0.05.

## Results

### 3.1 The antimicrobial activity of melatonin and ramelteon against Porphyromonas gingivalis planktonic culture

**Fig 1:**
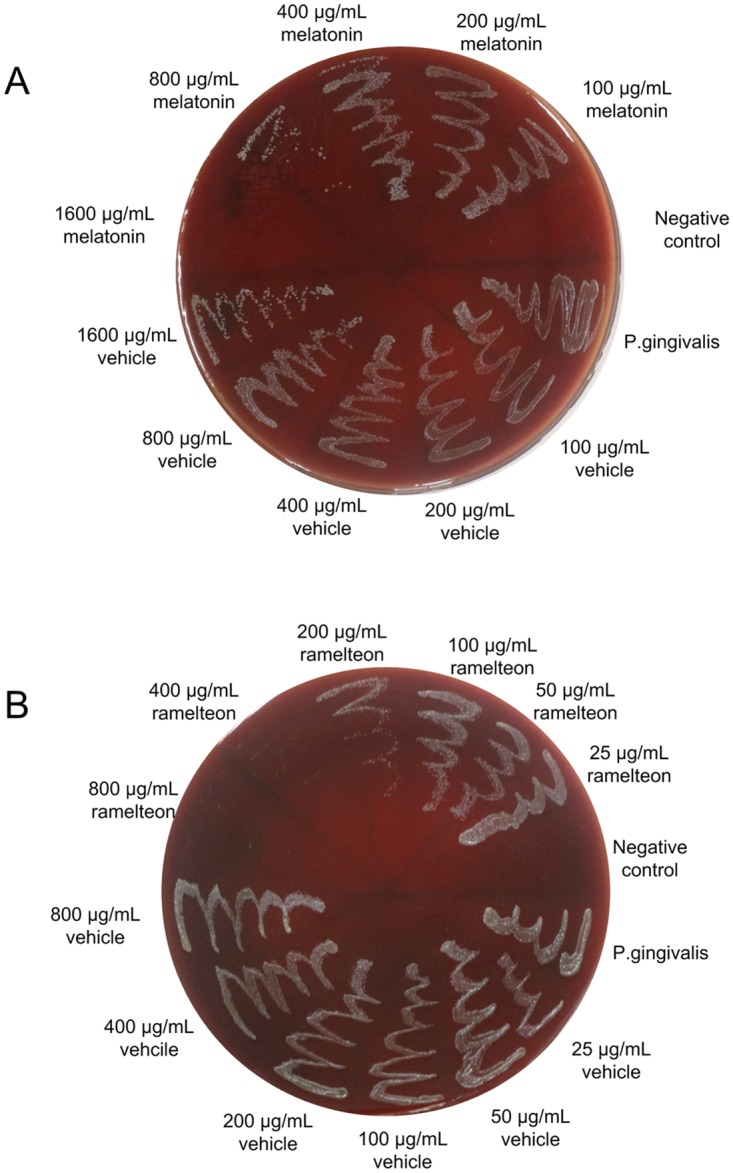
The number of colonies after 48 h-incubation of *Porphyromonas gingivalis* in blood agar. (A) The top six areas represent melatonin-containing wells; the bottom six areas are vehicle (DMSO) only-containing wells. (B) The top six areas represent ramelteon-containing wells. The bottom six areas are vehicle (DMSO) only-containing wells. More than three individual experiments were used for the measurement.

Results of the susceptibility assay of Porphyromonas gingivalis planktonic cultures to melatonin and ramelteon are summarized in Table 2. Both drugs showed a dose-dependent inhibition on in vitro growth of Porphyromonas gingivalis (Fig 1). As shown in Table 2 and Fig 1, the MIC and MBC of Melatonin were 100 μg/mL and 1600 μg/mL, respectively, while those of ramelteon were 50 μg/mL and 400 μg/mL, respectively.

**Table 2:** Antimicrobial effect of melatonin and ramelteon against P. gingivalis planktonic and biofilm.

Drug	Planktonic P.gingivalis		P.gingivalis biofilm		
	MIC(μg/mL)	MBC(μg/mL)	Formation	Reduction	Viability
			MBIC_50_ (μg/mL)	MBRC_50_ (μg/mL)	SMIC_50_ (μg/mL)
Melatonin	100	1600	50	200	100
Ramelteon	50	400	25	100	50

More than three individual experiments were used for the measurement.

### 3.2 Effect of melatonin and ramelteon on Porphyromonas gingivalis biofilms

**Fig 2:**
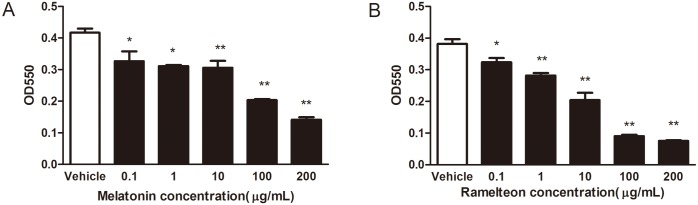
Effect of melatonin and ramelteon on *Porphyromonas gingivalis* biofilms formation. *Porphyromonas gingivalis* were incubated in the presence of melatonin (A) and ramelteon (B). After crystal violet staining, absorbance at 550 nm was recorded. Data represent the mean ± SEM (*n* = 3) and differences between groups were assessed by ANOVA, *P < 0.05 versus vehicle control; **P < 0.01 versus vehicle control. three experiments.

In addition to growth inhibition of *Porphyromonas gingivalis* planktonic culture, melatonin and ramelteon also noticeably showed antimicrobial activity against *Porphyromonas gingivalis* biofilms. Biofilm formation by *Porphyromonas gingivalis* considerably decreased in response to melatonin and ramelteon in a dose-dependent manner (Fig 2). As shown in Table 2, the MBIC_50_ values of melatonin and ramelteon were 50 μg/mL and 25 μg/mL, respectively. Furthermore, the mature biofilm attached at the bottom of the microplate for 48 h was reduced by melatonin (MBRC_50_ = 200 μg/mL) and ramelteon (MBRC_50_ = 100 μg/mL). In addition, melatonin and ramelteon decreased biofilm metabolic activity, suggesting that the bacterial cells’ viability within the biofilm was inhibited by the drugs (SMIC_50_ = 100 μg/mL for melatonin; SMIC_50_ = 50 μg/mL for ramelteon).

**Fig 3:**
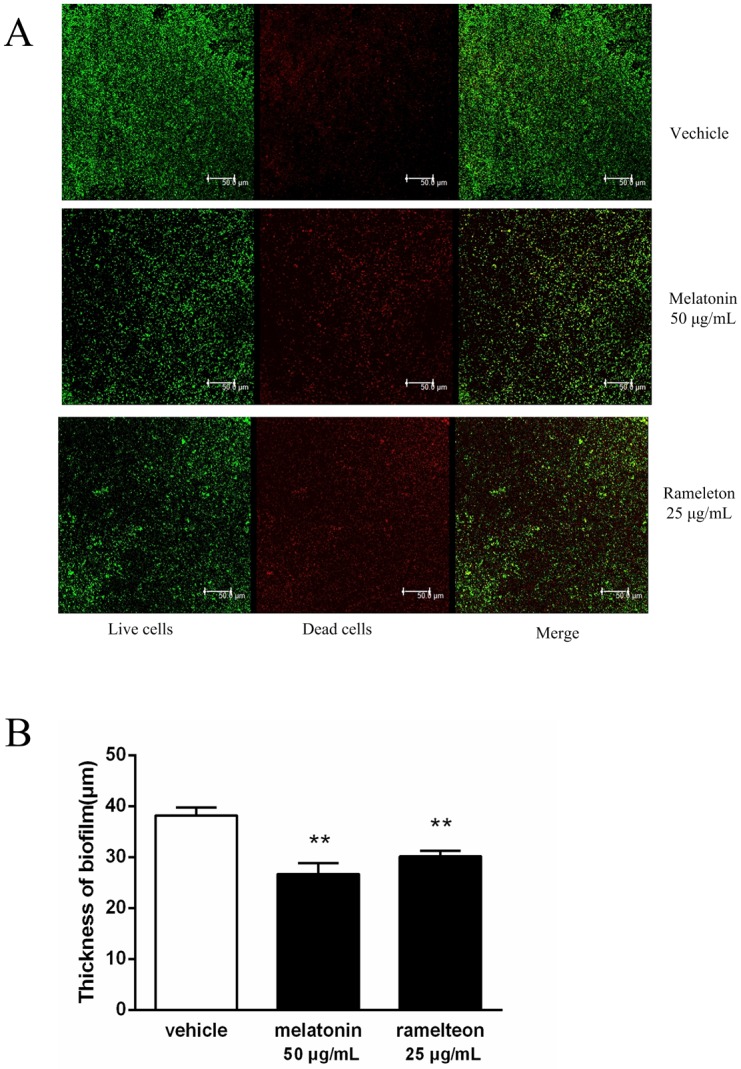
CLSM observation of *Porphyromonas gingivalis* biofilms formed in the presence of melatonin and ramelteon at sub-MIC concentrations. (A) CLSM images of *Porphyromonas gingivalis* biofilm formed in the presence of melatonin and ramelteon at sub-MIC concentrations. Biofilm-forming cells were stained using the Live/Dead Bacterial Viability Kit. Dead cells were stained red, whereas live bacteria were stained green. In the presence of 50 μg/mL or 25 μg/mL ramelteon, the areas of biofilm formation were narrower than that of vehicle controls. Bars = 50 μm. (B) Effects of melatonin and ramelteon on the thickness of *Porphyromonas gingivalis* biofilms. Data represent the mean ± SEM (*n* = 12) and differences between groups were assessed by ANOVA, **P < 0.01 versus vehicle control.

To further determine the effect of melatonin and ramelteon at sub-MIC concentrations on biofilm formation, 2-day-old biofilms of *Porphyromonas gingivalis* were observed by CLSM. The biofilms were made up of viable Porphyromonas gingivalis (Fig 3). When treatment of 50 μg/mL melatonin or 25 μg/mL ramelteon, there were much more dead cells observed in biofilms compared to the control group. The volumes of *Porphyromonas gingivalis* biofilms with 50 μg/mL melatonin or 25 μg/mL ramelteon were smaller and thinner than that of the control (Fig 3A). In the control biofilms, *Porphyromonas gingivalis* cells aggregated clusters. Conversely, bacterial cells were interspersed in melatonin- and ramelteon-treated biofilms. In addition, the average thicknesses of biofilms were determined by CLSM (Fig 3B). The thicknesses of melatonin-treated biofilms and ramelteon–treated biofilms were 26.7 ± 2.2 μm and 30.1 ± 1.1 μm, respectively, which were thinner than the control group (38.2 ± 1.5 μm). These data demonstrated that melatonin and ramelteon inhibited biofilm formation, reduced the established biofilms, and decreased biofilm viability.

### 3.3 Effect of melatonin and ramelteon at sub-MIC concentrations on Porphyromonas gingivalis gingipain activities

**Fig 4:**
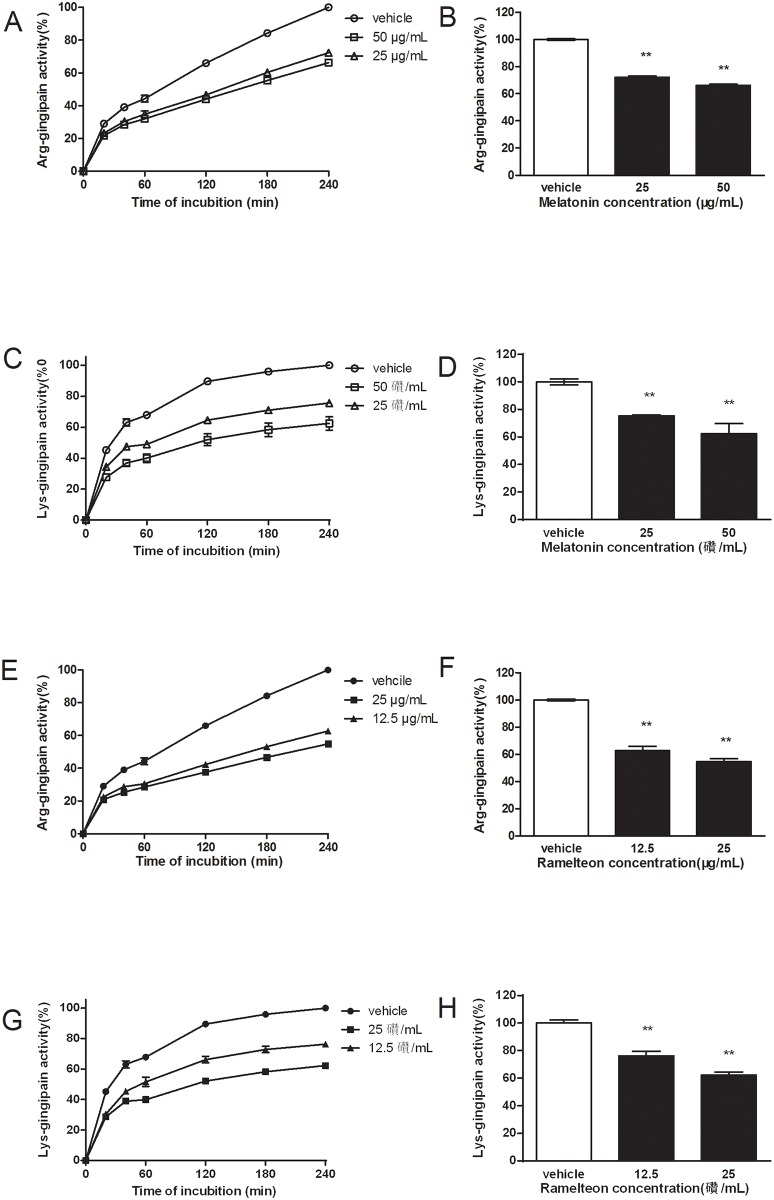
Effect of melatonin and ramelteon at sub-MIC concentrations on *Porphyromonas gingivalis* Rgp and Kgp activities. A 100% value was assigned to the degradation obtained after a 4 h-treatment in the absence of drugs. A, B, E, and F show the Rgp activities. C, D, G, and H show the Kgp activities. The inhibition of substrate degradation was also measured as a function of time (B, D, F, H). Data represent the mean ± SEM (*n* = 3) and differences between groups were assessed by ANOVA, **P < 0.01 versus vehicle control.

The activities of Rgp and Kgp by melatonin at sub-MIC concentrations were measured. Melatonin inhibited Rgp and Kgp activities in time-dependent and dose-dependent manners. After a 4-hour incubation, significant inhibition was observed. The inhibition rates by 25 μg/mL melatonin on Rgp and Kgp activities were 27.7% and 33.8%, respectively (Fig 4B and 4D). The inhibition rates of 50 μg/mL melatonin on Rgp and Kgp activities were 24.5% and 37.6%, respectively. The inhibitory effects within 4 hours are also shown in Fig 4A and 4C.

The activities of Rgp and Kgp were also inhibited by ramelteon at sub-MIC concentrations (p < 0.001). As shown in Fig 4F and 4H, the inhibition rates of 12.5 μg/mL ramelteon on Rgp and Kgp activities were 37.3% and 45.2%, respectively. The inhibition rates of 25 μg/mL ramelteon on Rgp and Kgp activities were 23.8% and 37.8%, respectively. The inhibitory effects within 4 hours are also shown in Fig 4E and 4G.

### 3.4 Effect of melatonin and ramelteon at sub-MIC concentrations on hemolytic activity

**Fig 5:**
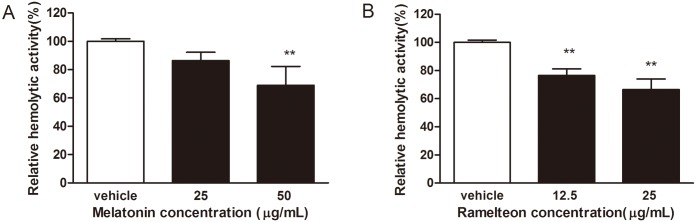
Effect of melatonin and ramelteon at sub-MIC concentrations on *Porphyromonas gingivalis* hemolytic activity. As a negative control, erythrocytes were used alone. Relative hemolytic activity was determined compared to the vehicle group. The hemolytic activity of vehicle-treated *Porphyromonas gingivalis* was normalized as 100%, and the melatonin-treated group (A) and melatonin-treated group (B) reported here are shown as a percentage of the vehicle control. Data represent the mean ± SEM (*n* = 6) and differences between groups were assessed by ANOVA, **P < 0.01 versus vehicle control.

Because lysed erythrocytes by bacteria could release hemoglobin, the absorbance of 405 nm for red pigment was used to describe the hemolysis of erythrocytes. After a 4-hour incubation of a mixture of *Porphyromonas gingivalis* cells and 1% sheep erythrocytes, hemolytic activity of *Porphyromonas gingivalis* was suppressed by melatonin and ramelteon at sub-MIC concentrations. As shown in Fig 5A, the relative hemolytic activities of melatonin (25 and 50 μg/mL) were 86.32 ± 2.63% and 68.96 ± 5.44%, respectively, compared to the control. The relative hemolytic activities of ramelteon (12.5 and 25 μg/mL) were 76.571 ± 2.28% and 66.56 ± 3.36%, respectively, compared to the control’s activity (Fig 5B). The results indicated that melatonin and ramelteon at sub-MIC concentrations inhibited the hemolytic activity of *Porphyromonas gingivalis*.

### 3.5 Effect of melatonin and ramelteon at sub-MIC concentrations on mRNA expression of virulence factors in Porphyromonas gingivalis

**Fig 6:**
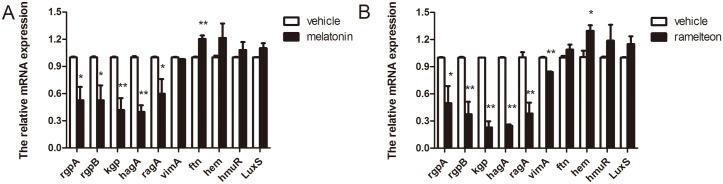
Effect of melatonin and ramelteon at sub-MIC concentrations on mRNA expression of virulence factors and iron/heme utilization-related genes in *Porphyromonas gingivalis*. Bacteria were incubated in the presence of 50 μg/mL melatonin (A) and 25 μg/mL ramelteon (B). Data are expressed as the mean ± SEM (*n* = 4) and differences between groups were assessed by ANOVA. The expression was normalized to *galE*. *P < 0.05 versus vehicle control. **P < 0.01 versus vehicle control.

The transcriptomic responses of virulence factors to melatonin and ramelteon were evaluated using real-time PCR analysis. As shown in Fig 6A, the expression of virulence factors, including *rgpA*, *rgpB*, *kgp*, *hagA*, and *ragA*, was noticeably inhibited, while the expression of ftn, which encodes an iron/heme utilization-related protein, significantly increased in the presence of 50 μg/mL melatonin. In the presence of 25 μg/mL ramelteon, the expression of virulence factors, including *rgpA*, *rgpB*, *kgp*, *hagA*, *ragA*, and *vimA*, also significantly decreased, while the expression of hem, which encodes an iron/heme utilization-related protein, significantly increased (Fig 6B). The expression of *hmuR*, which encodes a heme iron metabolism-related protein, did not significantly change, although it did show a non-significant, slight increase. The results indicate that melatonin and ramelteon inhibited the expression of virulence factors and affected the expression of iron/heme utilization-related genes in *Porphyromonas gingivalis*.

### 3.6 Cell viability and anti-inflammation of melatonin and ramelteon on HGFs

**Fig 7:**
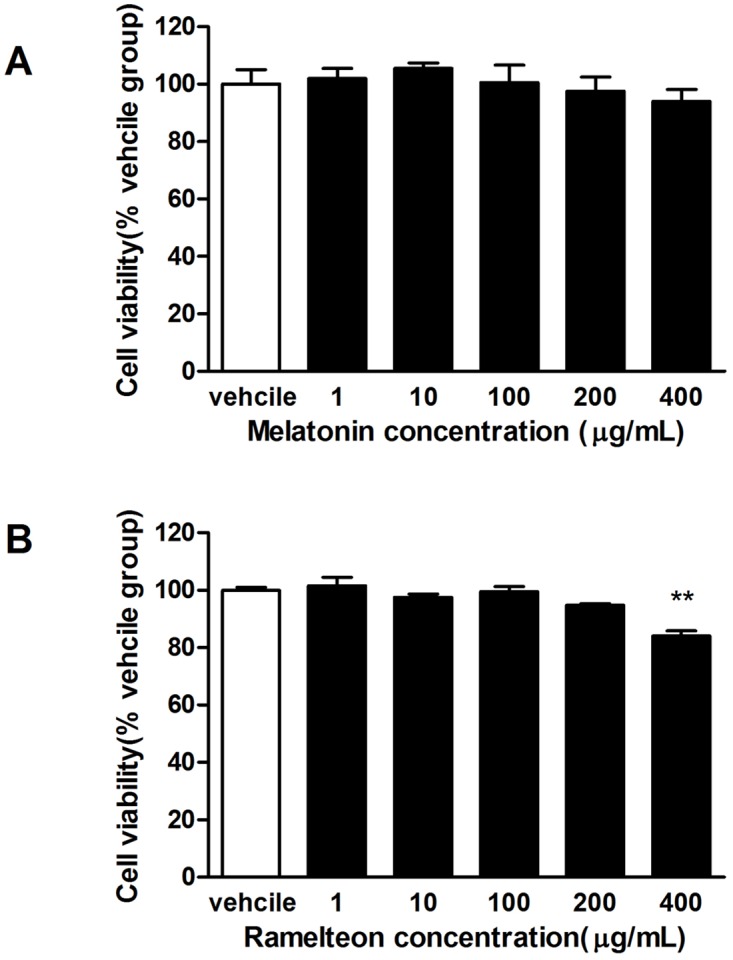
Effect of melatonin and ramelteon on HGFs viability. Treatment with melatonin has no obvious cytotoxicity on HGFs using MTT. Treatment with ramelteon up to 200 μg/mL has no significant cytotoxicity on HGFs. The cell viability obtained in the presence of the vehicle was assigned a value of 100%. Data represent the mean ± SEM (*n* = 4) and differences between groups were assessed by ANOVA, **P < 0.01 versus vehicle control.

The cytotoxicity of melatonin and ramelteon on HGFs was investigated using MTT. As shown in Fig 7, there were no obvious cytotoxic effects with a 24 h treatment with melatonin. Similarly, there were no obvious cytotoxic effects following a 24 h treatment with up to 200 μg/mL ramelteon.

**Fig 8:**
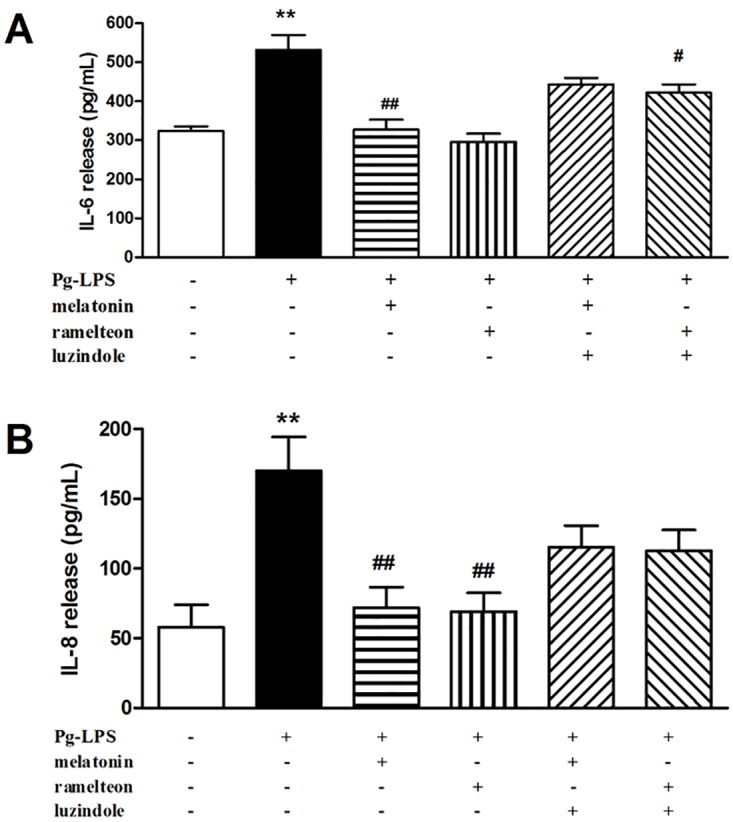
Effect of melatonin and ramelteon on the levels of IL-6 (A) and IL-8 (B) in HGFs. HGFs were treated with melatonin receptor agonists for 6 h, followed by Pg-LPS at 1 μg/mL concentration for 24 h. Data are expressed as the mean ± SEM (*n* = 4) and differences between groups were determined by ANOVA. **P<0.01 versus control. ^#^*P*<0.05 versus Pg-LPS group, ^##^*P*<0.05 versus Pg-LPS group.

As shown in Fig 8, the releases of IL-6 and IL-8 were stimulated by Pg-LPS after 24 h of exposure, 531.6±38.5 pg/mL and 156.1±23.5 pg/mL, respectively. Melatonin at the sub-MIC concentration (25 μg/mL) inhibited Pg-LPS-stimulated IL-6 and IL-8 production, 327.1±25.7 pg/mL and 72.2±11.7 pg/mL, respectively. Similarly, ramelteon at the sub-MIC concentration (12.5 μg/mL) also inhibited IL-6 and IL-8 production to a great extent, 296.4±20.7 pg/mL and 69.3±13.3 pg/mL, respectively. Furthermore, the protective effects of melatonin and ramelteon were reversed by the melatonin receptor antagonist luzindole (100 nM) (Fig 8). The above data demonstrated that melatonin receptor agonists inhibited Pg-LPS -stimulated cytokines expression, which was reversed by the melatonin receptor antagonist.

## Discussion

This study reports the effects of melatonin and melatonin receptor agonist on the key periodontal pathogen *Porphyromonas gingivalis* for the first time. We show that they have in vitro antimicrobial activity against *Porphyromonas gingivalis*, including the inhibition of biofilm formation, reduction of established biofilms, suppression of Kgp and Rgp, and inhibition of hemolytic activity. The effects may be associated with the regulation of virulence factor genes and iron/heme utilization-related genes. In the meantime, they also exert anti-inflammation from PG-LPS-stimulated cytokines release via melatonin receptor. These data preliminarily indicate that melatonin receptor agonists may be as novel “perioceutics” agents that effectively exert antimicrobial activity and anti-inflammation.

Oral bacteria are associated with highly organized microbial communities in the form of dental plaques [42]. As far as most oral pathogenic species are concerned, one of the most key virulence factors is the ability to form biofilms. Bacteria within a biofilm are less sensitive to antimicrobial agents than their planktonic counterparts and exhibit insensitivity to host defense systems [43–45]. So, we studied the effects of melatonin and ramelteon on *Porphyromonas gingivalis* biofilms, based on MIC and MBC results suggesting that they inhibit planktonic bacteria. Furthermore, both melatonin and ramelteon show inhibitory effects on *Porphyromonas gingivalis* biofilm formation and established biofilms. The inhibition of established biofilm formation may largely contribute to the reduction of bacterial growth and increased cell death. Another explanation may be due to the damage of the organized microstructure in *Porphyromonas gingivalis* biofilms because melatonin and ramelteon are of high lipophilicity and the ability to enter membranes, as disintegrated biofilms are more vulnerable to drug treatment.

It is well known that Rgp and Kgp are predominant extracellular proteolytic enzymes and key virulence factors of *Porphyromonas gingivalis* [46]. They play a pivotal role in the pathogenic factors of *Porphyromonas gingivalis*, for example fimbriae assembly and the processing of outer membrane proteins, and degradation of host proteins. Some of host proteins are thoroughly digested into peptides for furnishing *Porphyromonas gingivalis* growth and metabolism. The others are proteolyzed limitedly or selectively, resulting in the impairment of host immune defense and incapable of avoiding *Porphyromonas gingivalis* [34, 47–49]. So, as far as the above factors are concerned, the suppression of bacterial gingipains by melatonin and its derivative may benefit their potential application in the therapy of periodontitis. Our data showed that sub-MIC concentrations of melatonin and ramelteon could significantly inhibit the hydrolytic activities of *Porphyromonas gingivalis* gingipains. The inhibition of gingipains by melatonin and ramelteon will also deprive *Porphyromonas gingivalis* of available iron and prevent some tissue destruction. Thus, it suggested that these substances may be beneficial to reduce periodontal tissue destruction through the proteinase activity of *Porphyromonas gingivalis*.

Besides a broad spectrum of proteolytic enzymes in vitro, *Porphyromonas gingivalis* also produces hemolysin, which functions in the release of heme from erythrocytes. These enzymes likely degrade host tissues and secretions for the metabolism [38]. The release of heme from the hemoglobin molecule during the in vivo course of inflammatory periodontal disease may be a major consequence of this disease, and it may control the growth of *Porphyromonas gingivalis* in the subgingival environment [50]. It demonstrated that *Porphyromonas gingivalis* hemolysin is capable of degrading the hemoglobin molecule and eventually transporting it into the cell [51]. The mechanism of hemin acquisition from erythrocytes involves hemagglutination, hemolysis, binding, and degradation of the hemoglobin molecule [51]. Due to its high metal binding capacity, melatonin is assumed to function differently from known antimicrobials by reducing intracellular substrates in microorganisms [52]. In our study, melatonin and ramelteon inhibited hemolytic activity and may be associated with metal binding.

In addition, genes involved in virulence of *Porphyromonas gingivalis* were affected by treatment with melatonin and ramelteon. Melatonin and ramelteon inhibited the expression of *kgp*, *rgpB*, *vimA*, *ragA*, and *hagA*, while they increased the expression of *hmuR*, *luxS*, *ftn*, and *hem*. HagA is considered to be a key virulence factor because of the acquisition of heme from erythrocytes and other host cells, necessarily for bacterial growth [53–55]. HagA is also involved in attachment to human coronary artery endothelial cells and gingival epithelial cells [53–55]. RagA, which is the immunodominant surface antigen in the serum of patients with periodontal disease, is stimulated when *Porphyromonas gingivalis* is treated with cotinine, nicotine, and cigarette smoke extract. Rag is the homolog of TonB-linked outer membrane receptors and involved in the acquisition of iron in *Porphyromonas gingivalis* [55–57]. Rgp B plays a critical role in the periodontal disease because it is in charge of the production of gingipains [55,58,59]. Consistent with the above result, the above virulence-related genes were suppressed by melatonin and ramelteon, which may play a role in their antimicrobial and antiprotease activities.

In our study, the gene *ftn* was up-regulated by melatonin and *hem* by ramelteon. Although there were not significant differences in the expression levels of *hmuR* and *luxS*, the drugs had a tendency to increase the expression levels of genes related to iron/heme utilization. Indeed, there exists a relationship between iron/heme uptake and virulence in *Porphyromonas gingivalis* [60]. However, the virulence factors of *Porphyromonas gingivalis* are very complicated and multifactorial, so the nature of this regulation is still unclear. This inconsistency requires further study.

Due to their lipophilic nature, melatonin and ramelteon can easily access to the cell. They directly deposit in the oral cavity so that they have the high concentration to improve the health of oral tissues. One of the desired properties of melatonin receptor agonists is low toxicity to eukaryotic cells. In this study, the MTT assay was conducted with HGFs to assess cytotoxicity. Melatonin had no obvious cytotoxic effects, which was consistent with previous results that examined the proliferation of HGFs and MC3T3-E1 cells [8,16]. Similarly, no obvious cytotoxic effects were observed following a 24h-treatment with up to 200 μg/mL ramelteon.

PG-LPS, a component of the gram-negative bacterial cell wall, has been identified as the main virulence factor to induce periodontal disease and in correlation with the severity of periodontal disease [61,62]. It can stimulate the production of interleukins (IL), such as IL-6 and IL-8, in HGFs [26,63–65]. IL-6 is the inflammatory cytokine produced after microbial recognition and is involved in the pathogenesis periodontal disease, such as osteoclast formation, bone resorption, and periodontal destruction. IL-8 can recruit neutrophils to the periodontal lesion as a major chemoattractant. These ILs promote the degeneration of inflamed periodontal tissues and inhibition of them should be beneficial to treat periodontal disease [65,66]. In the present study, melatonin and ramelteon at sub-MIC concentration were both found to inhibit IL-6 and IL-8 production. The anti-inflammatory action is partly through melatonin receptors because this action was reversed by the melatonin receptor antagonist luzindole. It has been proved anti-inflammatory action of melatonin is thought as a direct result of potent radical-scavenging ability via the MT receptor [67,68]. It has also been reported that ramelteon reduced nicotinamide adenine dinucleotide phosphate (NADPH) partly mediated by MT receptor, which is involved in oxidative stress [69]. Based on these reports, we believe that anti-inflammation of melatonin and ramelteon may have involved an antioxidant effect partly caused by MT receptor.

In conclusion, this study firstly showed that melatonin and ramelteon have in vitro antimicrobial activity against both the planktonic culture and biofilms of *Porphyromonas gingivalis*. They inhibit the proteinase activities of gingipain and hemolytic activity, which are associated with the expression of virulence factors and iron/heme utilization. Furthermore, they exhibit anti-inflammatory properties via melatonin receptors. Our study provides new evidence that melatonin receptor agonists might be useful as the novel “Perioceutics” agents in periodontal disease therapy.
